# Opioid dependency rehabilitation with the opioid maintenance treatment programme - a qualitative study from the clients’ perspective

**DOI:** 10.1186/s13011-015-0031-4

**Published:** 2015-09-15

**Authors:** Arild Granerud, Helge Toft

**Affiliations:** Hedmark University College, NO-2418 Elverum, Norway; Innlandet Hospital Trust, Divisjon Psykisk Helsevern, Postboks 104, NO-2381 Brumunddal, Norway

**Keywords:** Opioid maintenance treatment programme, User participation, Drug addiction, Mental health

## Abstract

**Background:**

Opioid maintenance treatment (OMT) is the most widely used treatment for opioid dependence. The opioid maintenance treatment (OMT) programme represents an opportunity for people who are opioid users to minimize the many negative health and societal outcomes associated with opioid use through meeting the physiological need of their bodies for opioids. The purpose of this study is to shed some light on how clients in the Norwegian OMT programme see their level of influence on their own treatment.

**Method:**

It is a qualitative enquiry using semi-structured interviews of seven OMT clients living in various locations in Norway. The analysis of the material utilized a grounded theory-inspired approach.

**Results:**

This study show that the clients who were part of the OMT programme had better lives than people with untreated addictions did. However, the participants experienced having to play by the rules of the OMT programme if they wanted to have successful treatment. This resulted in varying degrees of dissatisfaction with the treatment.

**Conclusions:**

The results indicated that the clients felt objectified and disenfranchised in the OMT programme, and points out the low level of influence on their own treatment felt by the OMT clients.

## Background

People who have used short-acting opiates, such as heroin or morphine-like drugs (opioids), for a long time will develop neurobiological changes to their endorphin system [1]. The opioid maintenance treatment (OMT) programme represents an opportunity for people who are opioid users to minimize the many negative health and societal outcomes associated with opioid use through meeting the physiological need of their bodies for opioids [2]. In Norway, substitution treatment for opioid addiction was implemented in 1998, and has existed in its current form since 2001 [3]. The model on which the Norwegian OMT programme is based originated in the USA in the 1960s. The results there showed that methadone rehabilitation enabled clients both to function at work and to reconnect with their families. However, the most striking finding was that the craving for heroin was removed [4, 5]. Treatment of opioid addiction with substitution therapy has shown an improved survival/increased survival rate, a reduction in health damage and a better quality of life than psychosocial treatment alone [6, 7]. The long half-life of methadone leads to a ‘narcotic blockade’, eliminating withdrawal symptoms for 24–36 h. Given in high doses, it reduces the craving for heroin and blocks the effect of injected heroin [8].

At the end of 2012, 7038 of Norway’s approximately 5 million inhabitants had received OMT treatment. The number of people on OMT treatment has increased every year since 1998, with the total number of people who had received OMT in Norway up to the end of 2012 being 9404 [9]. In other Nordic countries, such as Finland in 2009, 2400 people received opioid substitution therapy [10], whereas, in Sweden in 2008, 3395 patients received methadone or buprenorphine [11]. OMT is, for many, a lifelong treatment, but substitution drugs are also addictive [1]. We cannot exclude the possibility that drugs – methadone and buprenorphine – have side effects about which we have as yet no knowledge [12]. Methadone users have poorer cognitive functioning than others, e.g. a reduced ability to understand and perform everyday routines and problems absorbing verbal information [13]. Recent studies have shown buprenorphine-induced hepatitis caused by injection of buprenorphine and cardiovascular complications of high-dose methadone [14]. The rehabilitation programme within the Norwegian OMT programme results in limitation of clients’ freedom. The clients have to deal with control routines such as getting their daily drug dose at the pharmacy, taking it under observation and providing regular urine tests. If the client does not achieve sufficient control of his or her drug dependence, these measures are continued [1]. In addition, OMT recommends that the client have a collaborating group consisting of GPs, the OMT contact, and the social service to ensure good cooperation for the client to reach the goals for rehabilitation. A Norwegian study showed that there are large variations in interpretation of the national OMT guidelines, concerning choice of drug, dosage and duration of treatment, as well as interpretation of hospitalization and discharge criteria [15]. Investigating the views of people who are addicts about their healthcare treatment is essential to understand their needs. The better the healthcare services that people who are addicts receive, the better the results that we can expect from the treatment [16]. However, a national cross-sectional survey in England found that Drug Action Teams reported inadequate fundings and time pressure prevented appropriate support being provided to user involvement [17]. It is essential that health care providers acquire the resources and the support necessary for providing good user involvement. Clients who have good relationships with their therapists achieve better results after treatment: they use fewer drugs and are less involved in criminal activities because of a trusting and positive relationship with their therapists [18]. In Australia, a consumer participation initiative fostered a sense of clients and staff coming to know one another beyond the usual limitations of their relationship. Clients reported being viewed upon differently; as people rather than simply an identity category. For clients, the opportunity to have ‘a voice’ began to disrupt the routine objectification or dehumanisation. Having a voice was synonymous with having ones’ ‘humanness’ recognised [19]. Service flexibility and the responsiveness of treatment to clients’ needs are key components of high-quality opioid substitution treatment [20, 21]. OMT centres in Norway have limited knowledge about the satisfaction of their OMT clients with the treatment [9]. Therefore, it is important to get to know the OMT clients’ views on this treatment.

The aim of the present study was to develop a deep and detailed knowledge of the experiences of OMT clients and how this influences their own treatment.

### Client involvement

It is widely recognized that clients in need of health care, at all levels, should be empowered [22]. Involvement of the service users in their participation occurs in the relationship between professionals and clients. The lowest level of involvement is provision of information, the second highest level is client participation and the highest level is empowerment of the client. In the last power is transferred to the client [23]. Healthcare professionals should respect clients and take their symptoms and descriptions of suffering seriously [24]. Clients’ needs should decide what kind of treatment they get. The services must have a satisfactory quality and be given, in consultation with the clients, as individually adapted activities. In the UK, client involvement has been central to efforts to improve the quality of health services [25]. There is an attitude that professionals know best and clients of health services do not have sufficient knowledge to articulate realistically what kind of health care they need [23]. The Norwegian OMT guidelines emphasize that clients should experience client involvement [3]; this is repeated in the Patient and User Rights Act 1999. In the OMT, the power balance is particularly skewed because the doctor has final power over both an addictive agent and the control measures [15]. The healthcare system must be shaped by the clients’ needs [26], so the system must be adapted to the client, not the other way around. One way to achieve this is to gather together the clients’ expertise, information and experiences. Clients have stated that, in general, they need more information about treatment and options [24]. For them, it is important not to be stuck in the patient role, but to be seen in the other roles that they may have. Allocation of power to clients (empowerment) is most relevant when working with disadvantaged groups and people who use drugs, so they deserve their consultants being aware of the empowerment perspective [27]. This can be a step towards the person who is using drugs being perceived as an equal citizen, not just a service user [25, 28]. A theory that can explain the positive and less positive experiences of OMT clients that influence their own treatment is the theory of a sense of coherence (SOC) [29]. SOC consists of the following components: comprehensibility, manageability and meaningfulness. Comprehensibility is here defined as the extent to which one experiences stimuli to which one is exposed as understandable. Manageability concerns the extent to which one feels that one has the necessary resources for the situation. Meaningfulness is related to the extent to which one thinks that the demands with which one is faced are worth the time and effort [29].

## Method

This exploratory descriptive study utilizes a grounded theory-inspired approach [30]. Grounded theory implies the systematic collection and interpretation of, as well as reflection on, data to make a theoretical construction of social processes [30–32]. The sampling of participants and the collection and analysis of data are recursive processes [33] generated from and grounded in the data; this method seeks to understand the subjective meaning of people’s own realities [33–35]. The method is particularly useful when one wants to explore social phenomena as seen from the perspective of the person concerned – in their natural context [36].

It was an open recruitment of informants through a local charity organization and a Facebook group. Fifteen people agreed to an interview. However, conducting the interviews was difficult for many of the persons who agreed to be interviewed. Therefore, it was in all seven OMT clients who were interviewed. The empirical material from the interview gave a sufficient for analysis and publication although saturation cannot be invoked. Three of them were recruited via an OMT user group on Facebook (‘LAR Nett’ in Norwegian), and were interviewed via Skype. Four of the interviewees were recruited via a NGO for people who have opioid dependence and other dependences in a city in eastern Norway. There was a good spread of geography (four different districts in Norway), gender and age, and many of the interviewees had been on the OMT programme for several years. Four women and three men were interviewed, giving a total of seven, as mentioned above. The age ranged from 26 to 49 years, with an average of 41 years. The demographic data are displayed in Table 1.

**Table 1:** Overview of the interviewees in the study of opioid maintenance treatment (OMT) programme

Gender	Total	Age (years)	Years in the OMT
Male	2	26–35	1–3
	1	46–55	15
Female	2	36–45	3–11
	2	46–55	8–12

The interviews were carried out using qualitative, semi-structured interviews. In each interview, some new questions were used to give the richest possible empirical material and come as close as possible to saturation [30].

In grounded theory, data collection and data analysis are carried out in parallel and combine an inductive and a deductive process [30, 36, 37]. The interviews were recorded digitally and transcribed verbatim by the second author. The constant comparative method comprised: (1) open coding, the analytical process (involving line-by-line analysis) through which concepts are identified, and their properties and dimensions discovered in the data; (2) writing memos about the concepts and comparing similarities and differences in the phenomenon under study; (3) selective coding, focusing the analysis around a core variable, and reconceptualising categories and indicators; and (4) sorting and writing up the memos. The theoretical coding provides the possibility of thinking in a theoretical way rather than in descriptive terms. The result of the analysis describes the core category characterizing the subjective meaning of people’s own realities.

### Ethical considerations

People on the OMT programme are in a vulnerable situation. Vulnerability and competence to consent were specifically assessed for each participant through a conversation especially to assess whether participants were in satisfactory mental state before the actual interview and throughout the interview situation of the interviewer, whom have training in mental health. The interviewees were recruited through an open invitation in a Facebook group for OMT interested individuals and a NGO to avoid any pressure to join. They received information about the study, and were sure that it was voluntary to participate and all data would be anonymized. All interviewees gave their written, informed consent. The study’s research ethics were evaluated by the Data Protection Officer (Norwegian Social Science Data Services). The research ethics of the World Medical Association’s Helsinki Declaration were followed. This pertained particularly to the principle of no harm and to protect the participants’ privacy [38].

## Results

The seven interviewees/clients had many stories to tell about the OMT regimen. The main category was ‘*A better life – if you follow the rules of the game’*, which can be related to all categories and subcategories, and was expressed by all interviewees. All the respondents indicated that life on an OMT regimen was better than life before inclusion on the OMT programme. They were all excited to go to the pharmacy and pick up their OMT drug, and thus stay healthy, avoid withdrawal and be able to use their energy for their everyday lives. As they received a free OMT drug they did not have to spend time and effort raising money for drugs. The relationship between the categories can be seen in Table 2: the findings presented in Table 2 are anchored by quotes from interviewees.

**Table 2:** Main category, categories and subcategories identified from interviews with clients on the opioid maintenance treatment programme

Main category	‘A better life – if you follow the rules of the game’			
Categories	To get your life back	The asymmetrical power balance	OMT as demotivator	A feeling of infringement
Sub-categories	A feeling of freedom	Lack of co-determination in OMT	Unsatisfactory follow-up	Stigma
	To build trust	Fear of punishment	Suspicion never stops	Not being believed
	Improved quality of life	Differences between OMT districts	The negative focus	

### To get your life back

On the whole, several of the interviewees were satisfied with their lives, and expressed an experience of freedom. They all said that the OMT programme saves lives and can provide a good opportunity for rehabilitation. Through the OMT programme clients have the opportunity to regain the kind of life that most people have, such as going to a restaurant with your partner, relaxing at home or engaging in leisure activities. Via the OMT drug, the illegal drug became more distant and participants felt more physically relaxed. The quote below shows the importance of experienced support:

*For me who wants to become totally drug free it is positive that with OMT you have a certain supervision. That there is a certain control and meetings with the responsibility group.*

Five of the seven interviewees were working or have other daytime activity: One had a paid job, whereas the others were engaged in voluntary work. They talked about the experience of building trust through engaging in work. Interviewees stressed the importance of the drug substitution regimen in order to be able to function at work, and that for each individual the drug should be the one that works best. Some interviewees felt that their opinion was taken into consideration when it came to changing both the type of drug (individualization) and its dosage. The building of trust was important.

Two of the interviewees emphasized that it is not solely the substitution drug that enables them to function well in everyday life and work. These two were very satisfied with the attention that they had received. The OMT programme was much more than just receiving a drug. It was important to have frequent coordinating team meetings with a doctor, nurse, social worker and OMT programme consultant. They had meetings every 2–3 months. One interviewee said that the ability to keep a job – an internship in a shop – boosted her self-esteem. The ability to function in a job gave her self-confidence:

*And then I got the necessary self-confidence and I dared to go out … and now I actually apply for proper jobs.*

The OMT programme is a life-saving programme. One interviewee said that he would have been dead if he had not been included in the programme. He was a heavy multi-drug user and had had several overdoses before he was included in the OMT programme.

The OMT programme helped the participants to stay clean. It made it easy to abolish the craving for drugs. To be included in the OMT programme and receive drug substitution meant that they could concentrate on getting their lives back on track. One of the interviewees said that he regarded himself as a better human being after joining the OMT programme and starting a Subutex (buprenorphine) regimen. He said that the OMT programme had helped him distance himself from drugs, and consequently he did not have to lie and steal. The craving for drugs was gone when he was using Subutex:

*Before it was the first thing you thought about when you woke up in the morning and the last thing you thought about before you went to bed, and the whole day centred around that. But now you wake up in the morning, put a pill under your tongue and get going, and the day is just like any other day. You don’t think about it 24 h a day, like you did before.*

Interviewees indicated that the OMT programme had given them more freedom, but that they were obliged to follow the OMT system to carry on with their collection schemes and their drug dose. Overall, they had improved quality of life. The interviewees were happy to get replacement drugs, but their satisfaction over dealing with the OMT framework varied. Even those who were most critical of the OMT programme – despite a feeling of powerlessness caused by the OMT system and its conditions – appreciated that it saves lives and that life without it would have been worse.

### The asymmetrical power balance

The OMT programme has power over the clients’ lives, and the strength and opportunity to build them up but clients were afraid that they could use the power to the contrary. The participants experienced a lack of co-determination in the OMT programme and thought that their opinions were not taken into account. Several interviewees feared sanctions if they were honest about their use on the side or if they did not deliver their urine samples on time. The OMT regimen that the clients encountered, the attitudes that were projected and the extent to which the programme locally followed the OMT guidelines or made its own rules depended on what part of the country they lived in. One of the interviewees felt that it had been decided in advance what was to be discussed in the coordinating team meetings. The fact that he had subjects that he wanted to discuss was not so interesting to the other participants in the meeting. Another interviewee described such meetings as being for ‘reprimand by the principal’. Another dreaded every meeting and every phone call, even though she knew that it would most probably go well every time:

*There they are sitting, the three people that somehow have power over me, sitting there and go through the things I’ve done and how they perceive me. I also feel that if there is something I want to say in the meeting, it is not always taken fully into account. As if they have decided in advance what will be said.*

*You have no influence over the treatment.*

Several interviewees emphasized that the OMT programme should provide several types of drugs. In addition to the current medications methadone, Subutex and Suboxone, there are other drugs that are used in some countries, e.g. Substitol and legal heroin [39, 40]:

*There is a reason why it is heroin that we use. It’s because it works best. It cures both anxiety and nerves and abstinence. It’s somehow the ultimate drug. I think that many of those who don’t succeed with OMT may have managed if they had received heroin instead.*

The OMT guidelines are interpreted differently in various districts, and this could have consequences if you have to move to another part of the country, or you lose your GP with whom you have a good relationship:

*In some places, suddenly everyone must use Suboxone. You feel that you are part of a group that some people feel they can do as they please with.*

Another difficulty for patients on the OMT programme was that use on the side might have consequences for them taking their OMT drugs with them when on holiday. The result may be that you adapt to the OMT system in a way that minimizes the negative consequences for yourself:

*You are afraid of punishment. So my attitude has always been: ‘Say as little as possible, and certainly nothing that can be used against you later on.’*

The general perception was that the OMT staff were kind and nice, but were well aware that they had all the power:

*… they even threaten to take away my methadone, so I immediately began to step down the pills … . You can’t call that collaboration … I don’t have much choice. If they take my methadone away from me, it is very likely that I will end up in my grave.*

### The OMT programme as a demotivator

There was a perception of unsatisfactory follow-up. The OMT clients experienced that the personnel focuses on the negative as use of legal drugs rather than the fact that all goes well in other areas. Some pointed out that they should give acknowledgment to the OMT clients who dared to sit in a coordinating team meeting and admit to taking other drugs. With a response like this, more people may dare to be honest:

*That’s a little special with OMT, they are very drug focused. It is not so important that you have bought an apartment or go to school or … . It’s like ‘Yeah, but have you been taking drugs?’ You can sit all alone at home and have anxiety and a terrible life, but clean urine samples. And then you are sort of clever!*

Even if interviewees were doing well in various arenas such as housing and social ones, the OMT programme tended to focus more on clean urine samples, the motivation to stay away from drugs and to what extent clients stick to the agreement. According to the clients, the follow-up was unsatisfactory.

One of the interviewees claimed that the OMT programme is not so good at providing information about what the client is entitled to. The interviewees meant that this could partly be explained by the fact that some of the staff had little or no relevant training for their job as an OMT consultant. Furthermore, it was pointed out that several of the OMT employees have little or no experience with drugs. One of the interviewees wished that the OMT employees were people with personal experience within the programme:

*In the responsibility groups, that everyone is a member of, there should be someone who has stopped abusing drugs and who has participated in OMT. This person may be called an employee with personal experience. Because those who have never been stoned don’t know what we’re talking about! They pretend that they do, but they don’t, you know!*

Despite several years in the OMT system, the clients expressed disappointment about the fact that the control measures and meetings for the treatment system never ended. They felt that suspicion never stopped. Even if it is the clients who have requested treatment to get clean, the interviews revealed that interviewees have the impression that the OMT staff think that clients have a hidden agenda:

*You feel like a criminal. It is as if they assume that you will try to trick and cheat. As if they think that after all you are just a junkie, so they cannot entirely trust you!*

### A feeling of infringement

The interviewees pointed out that in the public debate one occasionally hears terms such as ‘state drugs’ used about narcotic drugs funded by the state and ‘state addicts’ about people who receive these narcotic drugs (methadone, Subutex or Suboxone) in a rehabilitation programme funded by state. It can be particularly hurtful for OMT clients when employees have condescending attitudes. Some clients expressed a feeling of stigmatization. The following quotes from two interviewees illustrate this:

*There is not much that makes me angrier than to hear their condescending attitude!*

*I wish they would see us as individuals rather than to think that all OMT clients are dirty drug addicts who will do anything to get something to get high on.*

Several clients had felt hurt and distrusted by the staff on the OMT programme. One reason is that the OMT client may have the feeling of not being a regular client, but a drug addict – with the negative connotations that this term implies. You are defined as a client, but still have a sense of being treated differently from clients who are not addicts. This feeling is caused by several conditions described as degrading by the OMT clients. Several mentioned feelings of inferiority and stigmatization. Only clients who are addicts must deliver urine samples under supervision and pick up their medication at certain times. Several of the interviewees thought that it was unreasonable to bring in urine samples a couple of times a week, and one of them did not understand why for the rest of her life she would be expected to deliver urine samples at short notice:

*We’re still a bit like second-rate citizens. There are not many other patient groups who have to come on certain days and fetch their medication, and at any time may get a phone call and be ordered to submit a urine sample while someone watches them pee.*

## Discussion

This study summarizes the experience of being a patient within the Norwegian OMT regimen. It has shown that clinicians have taken decisions without client involvement. Clinicians are under increased pressure to focus on dialogue and to be the client’s advocate – in addition to establishing a more equal relationship. Professional capacity is based on the clinician’s ability to listen to and interact with the client in ways that give space for the client’s agenda. This kind of new professionalism may appear devoid of paternalism [41]. How do you get clients to focus on this kind of client participation? Client participation is based on the assumption that it is best for the clients to participate in their own treatment, so that they get the best possible treatment [26]. One might think that the OMT consultant would dare to relinquish his or her power only if he or she possessed adequate experience and knowledge in the field of opioid dependency and OMT clients. Knowledge about client interaction is probably key to increased client interaction. The empirical data in this study suggest that clinicians in OMT regime commonly use their force and power. Client interaction is often used as a positive term covering various practices [42]. The findings of this study indicate that there are variations in the interpretation of the OMT guidelines in the various districts. Furthermore, there are clear similarities to Bobrova’s [16] research about the quality of services that clients receive and what kind of results they expect to achieve from the treatment. Several of the interviewees in the present study made it clear that their relationship with the OMT consultant was crucial for the results after treatment, and this is consistent with the study carried out by Joe et al. [18].

Two of the interviewees stated that their opinion was taken into account as far as both choice of the OMT drug and the drug’s strength were concerned. This was part of the reason why they functioned relatively well both mentally and physically. Thanks to the OMT programme, clients could spend their energy on something sensible rather than trying to get hold of drugs, and this was seen as meaningful. These interviewees considered it meaningful (compare Antonovsky [43]) that the OMT programme enabled them to live fairly normal lives and thus feel less like drug addicts. Our interpretation is that sense of coherence [43] has grown and become stronger in spite of the limitations of rehabilitation program. It was not always easy to comprehend the control routines and sanctions of the OMT programme. But the motivation to receive Subutex and thus improve the quality of life was so high that they chose to comply with the OMT programme’s requirements so as not to lose the OMT drug or a good drug collection system at the pharmacy. The motivation of the meaningfulness component is crucial. When you have motivation, you show courage – even though you may not fully understand the situation [29]. There is a duality among clients and healthcare providers when discussing client interactions. On the one hand, it is necessary to try to bring down the number of controls and rules and, on the other, we need to maintain a certain level of control measures to ensure the success of the OMT programme. Clients must be able to participate and be involved in their own treatment, although health care providers need to ensure patients and communities are safe. Sometimes this means more restrictive access to certain medications. The policy recommendation should state that the focus should be more on the situation, exploring challenges and progress, rather than on whether the urine samples are clean at any time.

### A better life – if …

Several interviewees felt that they got a lot of support from their OMT consultant, that they had a say and were able to participate in their own treatment. As a result, the OMT system was perceived as being more comprehensible. According to Antonovsky [29], one has a high degree of comprehension if stimuli from the outside world are predictable, or they at least can be placed in a context so it is possible to understand what is happening. The findings showed an expressed desire to be considered in a similar way to other clients with chronic diseases. Perhaps the OMT regimen is so strict that clients do not manage to comply with all its rules. You could wonder how long you would have to deliver urine samples when you have been clean for several years. Perhaps we should focus instead on what the client has accomplished. One possible way in which representatives of the OMT programme can achieve this and increase clients’ comprehension may be to invest time and energy in their personal relationship with the client [16]. Several interviewees felt that trust worked as a resource for them. When they were trusted by their partners in the treatment system, as the months and years went by they were granted better drug collection arrangements. By lowering the requirements for the individual so that it was easier to comply with the regimen, the OMT client’s self-efficacy increased. One of the interviewees said that she felt that her OMT consultant’s support was ‘almost too good’. This finding shows that presence and support are conducive to good mental health. In other words, the interviewee felt that it was a good resource when the OMT consultant was a person whom she could trust [43]. Some informants also described bringing the partner or supervisor at work to the coordinating team meetings as a factor that made things easier. These meetings represented a large challenge for many of the clients. It can make it easier to cope with these meetings if clients bring a trusted person with them [29]. Should clinicians who are coordinating the team meetings possibly focus more on the future than on control and rules?

Detoxification of the OMT medication is not the prime objective of treatment. The prime objective is continued physiological and social stabilization. As yet, there is no validated medical cure for opiate addiction. Until a curative medication or a safe curative procedure has been developed, many of the patients may have to remain in treatment for the duration of their lives to avoid relapses, increased criminality, subsequent overdoses and death [8]. Why do we not try to detoxify clients on the OMT programme to a greater extent? Another point is that Norway does not provide heroin as a replacement drug, which one of the interviewees pointed out is a reason for some of the heaviest users having trouble dealing with the limits set by the OMT programme. This might be due to a summarized research showing that clients receiving heroin-assisted treatment find it easier to cope with the restrictions, and are also less likely to use drugs on the side [40]. One might think that the fact that the OMT programme offers only Subutex, Suboxone and methadone is part of the reason why the heaviest abusers do not cope well. The client does not receive the necessary resources to strengthen the SOC [29]. Policy makers to better OMT program should discuss these factors.

### Methodological considerations

The dependability of the present study was ensured by following recognized scientific methods of data collection and analysis [33]. The confirmability was strengthened by the fact that the findings have been presented to peers who, on the whole, could relate to the findings. The credibility was also strengthened by the fact that several interviewees have participated in the OMT programme for many years and cover a wide age range [33]. It is not impossible to generalize based on seven OMT clients, however feedback from OMT clients in presentations in several fora shows a high degree of recognition with the findings. It was not easy to recruit people for interview, but interviewees had important knowledge that is worth discussing. The credibility is also strengthened by the fact that one of the authors has been working in this field for several years and consequently has knowledge about opioid dependency and mental health. None of the authors has special connection with OMT treatment, but both have extensive experience in mental health and opioid dependency. Credibility was maintained by the fact that the information in the present study represents actual events and not the author’s own assumptions. Three of the interviewees were recruited via OMT Net’s group on Facebook. By recruiting via the internet only those who have access to the internet and probably a certain level of resources are reached. The other interviewees were recruited by contacting a charity organization, and for that reason the selection was done via “a third part”. The fact that the interviewees in this study come from different OMT centres contributes to a broader and probably greater transferability than if all of them lived in the same place and came from the same OMT centre.

## Conclusions

The findings in the present study show that clients of the OMT programme have a better life if they follow the rules. It is clear that those clients who do not have a final say in decisions affecting them, could feel insulted that control never stops. Clients of the OMT programme would probably have experienced a higher quality of life if, in addition to receiving the OMT drug, they had felt that they could have more influence on their treatment. This would have increased their sense of coherence [29]. The findings of the present study show that, although some clients experienced client involvement, it was perceived as absent by a large majority. An asymmetrical power balance means that the treatment system is perceived as demotivating and humiliating. The findings from the present study must be taken into account when the OMT programme is enhanced, and should be investigated on a larger scale.
